# Chirurgische Techniken in der Therapie des Basalzellkarzinoms – eine prospektive Untersuchung

**DOI:** 10.1007/s00105-020-04685-1

**Published:** 2020-09-15

**Authors:** Lukas Kofler, Hans-Martin Häfner, Claudia Schulz, Martin Eichner, Katrin Kofler, Saskia Maria Schnabl, Helmut Breuninger

**Affiliations:** 1 Universitätshautklinik, Eberhard-Karls Universität Tübingen, Liebermeisterstr. 25, 72076 Tübingen, Deutschland; 2 Institut für Klinische Epidemiologie und angewandte Biometrie, Eberhard Karls Universität Tübingen, Silcherstr. 5, 72076 Tübingen, Deutschland

**Keywords:** 3‑D-Histologie, Epitheliale Hauttumoren, Dermatochirurgie, Kürettage, Rezidivrate, 3D histology, Epithelial skin cancer, Dermatologic surgery, Curettage, Recurrence rate

## Abstract

**Hintergrund:**

Basalzellkarzinome sind die häufigsten epithelialen Hauttumoren und eine häufige Indikation für dermatologische Eingriffe. Trotz der Etablierung medikamentöser Therapieoptionen stellt die Operation weiterhin die Therapie der Wahl dar. Hierbei stehen verschiedene Möglichkeiten zur Verfügung, die von der Kürettage bis hin zu komplexen dermatochirurgischen Eingriffen reichen. Neben dem Hauptaspekt der geringen lokalen Rezidivraten sind auch ästhetische Faktoren und die Anzahl der Eingriffe für die Wahl der Therapie wichtig.

**Methoden:**

In dieser Studie wurden 347 Patienten mit 398 Basalzellkarzinomen (nodulärer Typ, Durchmesser bis 10 mm) prospektiv untersucht. Die Patienten wurden randomisiert in 2 Behandlungsarme eingeteilt: In einer Gruppe wurden die Tumoren kürettiert, in der anderen Gruppe exzidiert. Als Kontrolle dienten Patienten, die im gleichen Untersuchungszeitraum 3‑D-histologisch kontrolliert operiert wurden.

**Ergebnisse:**

Die höchste lokale Rezidivrate wurde nach der Kürettage (14,0 %) beobachtet, während die Gruppe mit 3‑D-Histologie die niedrigste Rezidivrate (0,9 %; *p* < 0,001) aufwies. In der 3‑D-Gruppe waren mehr Re-Exzisionen erforderlich, um eine vollständige Entfernung des Tumors zu erreichen, als in der Gruppe mit histologischen Serienschnitten. Die Patienten bewerteten das ästhetische Ergebnis am besten nach der Kürettage. Die mittlere Nachbeobachtungszeit betrug 3,9 Jahre.

**Schlussfolgerung:**

Die Wahl der chirurgischen Therapie bei kleinen nodulären Basalzellkarzinomen hängt von den individuellen Gegebenheiten ab. 3‑D-histologisch kontrollierte Exzisionen mit Wundverschluss nach vollständiger Tumorentfernung zeigten in unserer Studie die geringste Rezidivrate. Aber auch die Kürettage stellt eine mögliche chirurgische Therapieoption mit minimalem Aufwand und einer akzeptablen Rezidivrate dar, die zu guten ästhetischen Ergebnissen führen kann.

## Abstract (en)

**Background:**

Basal cell carcinomas are the most frequent epithelial skin tumors and a frequent indication for dermatological interventions. Despite the establishment of drug treatment options, surgery is still the treatment of choice. Various options are available ranging from curettage to complex dermatosurgical procedures. In addition to the main aspect of low local recurrence rates, esthetic factors and the number of procedures are also important for the choice of treatment.

**Methods:**

In this study 347 patients with 398 basal cell carcinomas (nodular type, diameter up to 10 mm) were prospectively examined. The patients were randomized into two treatment arms: in one group the tumor was treated by ring curettage and in the other group the tumor was excised. Patients who underwent 3D histologically controlled surgery due to basal cell carcinoma during the same investigation period served as controls.

**Results:**

The highest local recurrence rate was observed after curettage (14.0%), whereas the group with 3D histology had the lowest recurrence rate (0.9%, *p* < 0.001). In the 3D group, more re-excisions were required to achieve complete tumor clearance compared to the group who underwent excisions with serial section histology. Patients rated the esthetic outcome best after curettage. The median follow-up was 3.9 years.

**Conclusion:**

The choice of surgical treatment for small nodular basal cell carcinomas depends on individual circumstances. Excisions controlled by 3D histology with wound closure after complete tumor clearance showed the lowest recurrence rate in our study; however, curettage is also a possible surgical treatment option with minimal effort and an acceptable recurrence rate, which can lead to good esthetic results.

Basalzellkarzinome (BZK) stellen die häufigste Form von epithelialen Hauttumoren dar, wobei die Inzidenz in den letzten Jahren weiter zugenommen hat [14, 17, 34]. Daher sind BZK eine häufige Indikation für dermatochirurgische Eingriffe. Abhängig von der Lokalisation und dem Tumorsubtyp zeigt die mikrographisch kontrollierte Chirurgie erhebliche Vorteile [8]. Insbesondere im Gesichtsbereich ist durch ein mehrstufiges Verfahren eine erhöhte Sicherheit bei gleichzeitig verbesserten ästhetischen Ergebnissen möglich [6, 8, 10]. Neben der Mohs-Chirurgie, die mithilfe der Kryostattechnologie arbeitet, spielt die 3‑D-Histologie eine wichtige Rolle bei der chirurgischen Behandlung von epithelialen Hauttumoren [6, 8, 10]. Für die 3‑D-Histologie konnte gezeigt werden, dass im Vergleich zur Serienschnitttechnik deutlich mehr Tumorausläufer nachgewiesen werden können [5]. Die Operation stellt die effektivste Behandlung von BZK im Hinblick auf Rezidivfreiheit dar und gilt daher als die First-line-Therapie für diese Tumorentität [15, 25]. Das Lokalrezidivverhalten von BZK ist neben dem Tumorsubtyp und operativen Parametern in erster Linie mit der Art der histologischen Aufarbeitung assoziiert. Randomisierte Studien, die die histologische Aufarbeitung des BZK durch mikrographisch kontrollierte Chirurgie mit konventionellen Verfahren vergleichen, konnten jedoch keine kurzfristig höheren Rezidivraten für Letztere zeigen [20, 28].

Neben großen BZK oder schwierigen Lokalisationen gibt es auch eine beträchtliche Anzahl von frühen Tumoren oder BZK mit begrenzter Größe. In der Literatur wurde die chirurgische Therapie von frühen Formen des BZK wiederholt kritisch hinterfragt, und nichtchirurgische Therapieansätze ohne histologische Kontrolle wurden vorgeschlagen [32]. Für diese Tumorentität wurde sowohl die Anwendung von Immunstimulatoren als auch die photodynamische Therapie (PDT) beschrieben [2, 4, 9]. Diese Therapieoptionen zeigen lokale Rezidivraten von 16,0–40,0 % [15–17, 20, 22–25, 27–29]. Hier wurden sowohl Kollektive mit ausschließlich superfiziellen BZK [2, 23], superfiziellen und nodulären BZK [4, 29, 30, 35] oder ausschließlich nodulären BZK beschrieben [9, 36]. Die PDT zeigt die höchsten lokalen Rezidivraten in randomisierten Studien, wobei allgemein höhere Rezidivraten bei lokalen Therapien als bei operativen Verfahren beschrieben wurden [4, 9, 15].

Die Kürettage steht als minimal-invasiver chirurgischer Ansatz zusätzlich zur komplexen Exzision zur Verfügung, insbesondere für superfizielle BZK oder kleine noduläre BZK. Bei superfiziellen BZK des Rumpfes konnten gute Erfolge mittels Shave-Exzision gezeigt werden [1]. Im Rahmen von entzündlichen Prozessen, die auch nach der Kürettage auftreten, wurde eine Tumorregression von BZK beschrieben [12, 22, 31]. Vielmehr kann die Frage gestellt werden, ob kleine BZK oder frühe Formen dieses Tumors mit der mikrographischen Chirurgie sogar überbehandelt werden. Darüber hinaus spielen die Anzahl der erforderlichen Eingriffe und die damit verbundenen Risikofaktoren eine wesentliche Rolle in der klinischen Routine und sollten daher bei Patienten mit kleinen BZK sowie älteren Patienten in Betracht gezogen werden. Wie in der aktuellen Leitlinie dargestellt, soll sich das prinzipielle Behandlungsziel bei älteren Patienten mit BZK nicht von dem bei jüngeren Patienten unterscheiden. Tatsächlich ist in der Therapieplanung nicht so sehr das Alter in Jahren entscheidend, sondern Faktoren, welche häufig mit fortgeschrittenem Alter einhergehen. Für die Planung der Therapie von BZK sollten daher im Besonderen die Lebenserwartung von Patienten, weitere nichtkurative Therapieziele, vorhandene Komorbiditäten und Tumorcharakteristika berücksichtigt werden [7, 14, 19].

Eine prospektive Bewertung hinsichtlich des lokalen Rezidivverhaltens, der Anzahl der Reoperationen und des ästhetischen Ergebnisses nach Kürettage, kompletter Exzision und 3‑D-histologisch kontrollierter Komplettexzision scheint daher von allgemeinem Interesse zu sein. Dazu stellen wir eine randomisierte, kontrollierte Studie vor, in die Patienten mit histologisch gesicherten nodulären BZK und einem Tumordurchmesser von bis zu 10 mm eingeschlossen wurden.

## Material und Methoden

### Patienten

Die Studie wurde als randomisierte, kontrollierte Studie konzipiert, und Patienten, die zwischen Dezember 2008 und Januar 2010 in unserer Klinik aufgrund von histologisch gesicherten nodulären BZK mit einem maximalen Tumordurchmesser von 10 mm chirurgisch behandelt wurden, wurden eingeschlossen. Alle Patienten, bei denen histologisch ein anderer Subtyp vorlag, wurden aus der Analyse ausgeschlossen.

Die Randomisierung wurde in 2 Gruppen durchgeführt. Die erste Gruppe erhielt eine Kürettage („Kürettagegruppe“), und die anschließende histologische Auswertung wurde an einem repräsentativen Mittelschnitt des Exzidats durchgeführt. In der zweiten Gruppe wurde eine Exzision des Tumors durchgeführt, gefolgt von einer histologischen Untersuchung mittels Serienschnitten, auch bekannt als „Brotlaib-Technik“ („Serienschnittgruppe“). Um diese Ergebnisse in den Kontext des aktuellen Goldstandards zu setzten, wurden diesen Gruppen Patienten gegenübergestellt, die mit einer 3‑D-histologisch kontrollierten Exzision behandelt wurden („3-D-Gruppe“). Dazu wurden die Tumorränder und die Tumorbasis vom Chirurgen unmittelbar postoperativ in Histologiekassetten eingelegt, was eine exakte anatomische Zuordnung des Präparates ermöglichte. Dieses Vorgehen wurde gewählt, um die Kürettage bzw. Exzision mit anschließender Serienschnittevaluation dem Standardvorgehen unserer Klinik, der Exzision und Schnittrandkontrolle mittels 3‑D-Histologie, gegenüberzustellen. Planung und Umsetzung dieses Studiendesigns wurden gemeinsam mit der Abteilung für Medizinische Biometrie der Universität Tübingen erarbeitet und umgesetzt. Patienten, die in die 3‑D-Gruppe aufgenommen wurden, erhielten selbstverständlich ebenfalls eine Aufklärung und willigten in das Vorgehen ein. Die vorliegende Studie wurde von der Ethikkommission der Universität Tübingen genehmigt (Nr.: EK6/2006) und in Übereinstimmung mit den Richtlinien für gute klinische Praxis und der Deklaration von Helsinki ausgeführt.

Die histopathologische Untersuchung wurde von erfahrenen Dermatohistopathologen unserer Klinik durchgeführt. Die Randomisierung erfolgte durch das Institut für Klinische Epidemiologie und Angewandte Biometrie.

Alle Eingriffe wurden in einem voll ausgestatteten Operationssaal in Tumeszenzlokalanästhesie (TLA) durchgeführt. Die histologischen Untersuchungen wurden an paraffineingebetteten Schnitten durchgeführt.

Nach Kürettage wurde eine sekundären Wundheilung angestrebt, wobei SutureStrips plus (Derma Sciences, Plainsboro/NJ, USA) direkt auf die Wundfläche aufgeklebt wurden. Die Defekte in der Serienschnittgruppe sowie in der 3‑D-Gruppe wurden nach plastisch-chirurgischen Kriterien verschlossen. Hierfür wurden in Abhängigkeit von Größe, Lage und Form des Defektes Dehnungs‑, Verschiebe‑, Rotations- oder Transpositionslappenplastiken durchgeführt.

### Eingriffe

**Figure 1:**
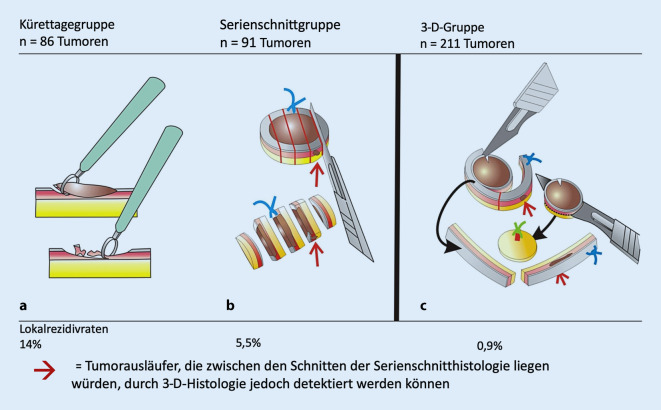

In der Kürettagegruppe wurde der Tumor mit einer 7‑mm-Ringkürette mit einem Sicherheitsabstand von 1–2 mm entfernt. Die Kürettage wurde so lange fortgesetzt, bis klinisch kein Tumor mehr zu sehen war; anschließend wurde das sichtbare Gewebe mit der Kürette im Sinne einer Sicherheitsnachexzision weiter entfernt (Abb. 1a). Das gewonnene Material wurde separat analysiert, indem ein repräsentativer Mittelteil des Gewebes nach Fixierung und Färbung (Hämatoxylin-Eosin) beurteilt wurde. Auch bei histologisch gesichertem Nachweis von Tumorresten wurden nach Zustimmung der Patienten keine Reexzisionen, sondern lediglich regelmäßige Nachkontrollen durchgeführt.

In der Serienschnittgruppe wurden die BZK mit dem Skalpell nach herkömmlichen Standards mit einem Sicherheitsabstand von 1–4 mm um den klinisch sichtbaren Tumor exzidiert. Die Wahl des Sicherheitsabstandes erfolgte in Abhängigkeit der Lokalisation sowie der klinischen Tumorgröße. Zur histologischen Beurteilung wurden vom Tumorpräparat parallele Serienschnitte von 2 mm Breite angefertigt (Abb. 1b). Anschließend wurden die Proben nach einem Standardprotokoll mit Formalin fixiert und abschließend gefärbt (Hämatoxylin-Eosin). Etwa 20 h später standen sie zur Untersuchung bereit. Wurden Tumoranteile im Randbereich oder in einem Abstand von weniger als 1 mm zu den Rändern gefunden, wurden erneute Exzisionen durchgeführt, bis die Tumorfreiheit der Ränder gewährleistet war.

Zur Kontrolle der Patienten in der Kürettage- und Serienschnittgruppe wurden auch Patienten erfasst, die im gleichen Zeitraum mit 3‑D-histologiekontrollierten Exzisionen behandelt wurden, da die 3‑D-Histologie für BZK in unserer Klinik als Routineverfahren etabliert ist. Diese Patienten wiesen ebenfalls histologisch gesicherte BZK von bis zu 10 mm auf und wurden separat dokumentiert (3-D-Gruppe). Das Follow-up dieser Patienten unterschied sich nicht von den anderen Gruppen. Auch in der 3‑D-Gruppe wurden die BZK mit einem Sicherheitsabstand von 1–4 mm exzidiert und anschließend vom Operateur in Histologiekassetten ausgerichtet. Dazu müssen die Ränder und die Basis gemäß klinikinternem Protokoll getrennt eingebettet werden (Abb. 1c). Es folgte eine Formalinfixierung und eine Hämatoxylin-Eosin-Färbung. Nachexzisionen wurden so lange durchgeführt, bis die R0-Resektion histologisch bestätigt wurde.

### Nachsorgeuntersuchungen

Alle Patienten erhielten 2‑mal jährlich eine Nachsorgeuntersuchung. Es erfolgten eine abwechselnde Nachuntersuchung und schriftliche Befragung hinsichtlich eines evtl. Tumorrezidivs. Als primärer Endpunkt der Studie wurde ein histologisch gesichertes Lokalrezidiv definiert. Im Falle eines Lokalrezidivs erhielten die Patienten routinemäßig eine 3‑D-histologisch kontrollierte Exzision.

Das ästhetische Ergebnis wurde nach 24 Monaten anhand von 5 möglichen Kategorien (exzellent, gut, befriedigend, mittel oder schlecht) beurteilt. Dabei wurde das ästhetische Ergebnis durch den Patienten selbst und nicht durch den jeweiligen Dermatochirurgen beurteilt, um eine Verzerrung der Ergebnisse zu vermeiden.

## Ergebnisse

Insgesamt wurden 347 Patienten mit 398 Tumoren ausgewertet (Tab. 1). Die untersuchten Gruppen zeigten keine signifikanten Unterschiede hinsichtlich des Nachbeobachtungszeitraums, Alters, Geschlechts, der Tumorgröße sowie des durchschnittlichen Sicherheitsabstands. Die überwiegende Mehrheit der Tumoren (93,0 %) befand sich im Kopf- und Halsbereich.

**Table 1:** 

	Kürettagegruppe	Serienschnittgruppe	3‑D-Gruppe
Patienten	74	78	195
Alter, Median (min./max.)	68,4 Jahre (min. 41/max. 93)	68,2 Jahre (min. 27/max. 89)	72,0 Jahre (min. 22/max. 98)
Geschlecht männlich/weiblich	54 %/46 %	54 %/46 %	49 %/51 %
Anzahl Tumor	86	91	221
Durchschnittlicher Tumordurchmesser (Standardabweichung; 95 %-Konfidenzintervall)	7,6 mm (3,2 mm; 7,0–8,2 mm)	7,6 mm (2,9 mm; 7,1–8,1 mm)	7,5 mm (2,7 mm; 7,1–7,9 mm)
Durchschnittlicher Sicherheitsabstand (min./max.)	–	2,1 mm (2,0–2,3 mm)	2,3 mm (2,2–2,4 mm)
Nachsorgezeitraum in Jahren, Median (min./max.)	3,9 (3,5–4,3)	3,6 (3,4–3,7)	3,9 (3,8–4,1)
Lokalrezidive, *n* (%)	12 (14 %)	5 (5,5 %)	2 (0,9 %)
Zeitraum bis Lokalrezidiv, Median	1,4 Jahre	2,2 Jahre	2,8 Jahre
Nachexzisionen (%)	0 (0 %)	16 (17 %)	54 (24 %)
2. Nachexzisionen (%)	0 (0 %)	0 (0 %)	10 (5 %)
R1-Resektion, *n* (%)	17 (19,8 %)	4 (4,4 %)	3 (1,4 %)
Lokalrezidive nach R1-Resektion, *n* (%)	4 (24 %)	1 (25 %)	0 (0 %)

**Figure 2:**
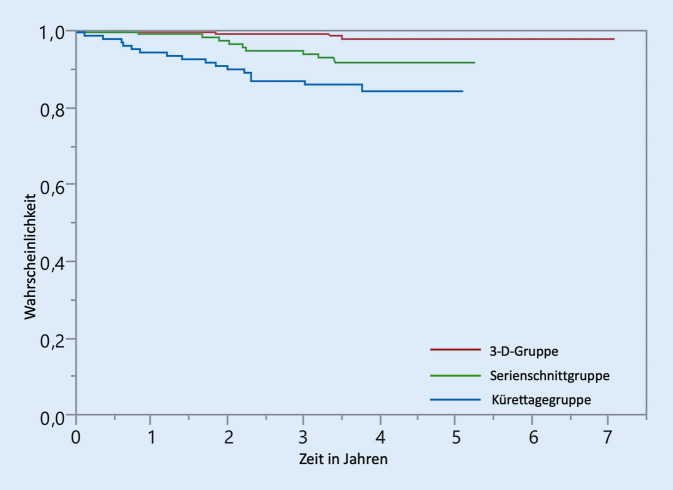

Die Gesamtlokalrezidivrate aller Tumoren in den Gruppen betrug 4,8 % (19/398). Allerdings zeigten sich in den einzelnen Gruppen deutliche Unterschiede (Tab. 1): In der Kürettagegruppe wurde bei 14,0 % der Patienten ein Lokalrezidiv gefunden (12/86), während in der Serienschnittgruppe im Untersuchungszeitraum nur 5,5 % Lokalrezidive verzeichnet wurden (5/91; *p* = 0,005). Das Lokalrezidiv in der Kürettagegruppe trat deutlich früher auf als in der Serienschnittgruppe (1,4 Jahre vs. 2,2 Jahre; Abb. 2). Patienten, die mit einer 3‑D-histologisch kontrollierten Operation behandelt wurden, zeigten eine signifikant niedrigere Lokalrezidivrate (0,9 %; *p* = 0,006) im Vergleich zur Serienschnittgruppe. Außerdem traten die Lokalrezidive in der 3‑D-Gruppe später auf als in den anderen Gruppen (2,8 Jahre; Abb. 2). Auch zwischen der Kürettagegruppe und der 3‑D-Gruppe unterschieden sich die Lokalrezidive hochsignifikant (*p* < 0,001).

In der Kürettagegruppe zeigten 19,8 % der Tumoren eine primäre R1-Resektion (17/86). In der Serienschnittgruppe wurde bei 16,5 % der Tumoren eine R1-Resektion nach dem ersten Eingriff beobachtet (17/91). Bei den 3‑D-Histologie-kontrolliert exzidierten Tumoren wurde bei 24,4 % eine primäre R1-Resektion gefunden (54/221).

In der Serienschnittgruppe war nur eine Reexzision bei den anfänglich R1-resezierten Patienten notwendig, um den Tumor vollständig zu resezieren. In der 3‑D-Gruppe erfolgte eine exakte anatomische Zuordnung der in situ verbleibenden Tumoranteile mit entsprechend begrenzter Reexzision nach den Standards der 3‑D-Histologie. Bei 75,6 % der Tumoren in der 3‑D-Gruppe war nur eine einzige Reexzision erforderlich. Weitere Reexzisionen waren bei 4,5 % aller Tumoren erforderlich (10/221).

In 1,4 % (3/221) zeigte sich trotz mikrographisch kontrollierter Reexzisionen eine R1-Situation als Endergebnis der chirurgischen Therapie. Die Ursache hierfür war der spezifische Wunsch der Patienten, keine weiteren Operationen durchzuführen. Dennoch zeigte keiner der R1-resezierten Patienten in der 3‑D-Gruppe ein Lokalrezidiv (0/3). Insgesamt zeigten 25,0 % (1/4) der R1-resezierten Tumoren in der Serienschnittgruppe und 23,5 % (4/17) der R1-resezierten Tumoren in der Kürettagegruppe ein Lokalrezidiv während des Untersuchungszeitraums.

Das ästhetische Ergebnis wurde am besten in der Kürettagegruppe bewertet, wobei 85,0 % der Patienten das Ergebnis als „exzellent“ oder „gut“ bewerteten. In der Gruppe der Serienschnitte wurde das ästhetische Ergebnis in 75,0 % der Fälle und in der 3‑D-Gruppe in 76,0 % mit „exzellent“ oder „gut“ bewertet.

Komplikationen wie Blutungen und Wundinfektionen wurden ebenfalls bewertet. In der Kürettagegruppe wurden mit 12,0 % am häufigsten Nachblutungen dokumentiert, während in der Serienschnittgruppe die Nachblutungsrate mit 9,0 % vergleichbar mit der 3‑D-Gruppe (8,0 %) war. Keiner der Patienten benötigte aufgrund von Nachblutungen eine Intervention. Lokale Wundinfektionen wurden in der Kürettagegruppe mit 3,0 % und in der Serienschnittgruppe und der 3‑D-Gruppe mit jeweils 4,0 % dokumentiert.

## Diskussion

In der vorliegenden Studie wurden die lokalen Rezidivraten nach verschiedenen chirurgischen Eingriffen sowie histologischen Aufarbeitungstechniken bei nodulären BZK mit bis zu 10 mm Durchmesser untersucht. BZK sind die häufigsten nichtmelanozytären Hauttumoren und zeigen eine zunehmende Inzidenz [14]. Da BZK aufgrund der UV-Assoziation insbesondere an stark Sonnenlicht-exponierten Körperstellen auftreten, wurden für die vorliegende Studie auch BZK im Gesichtsbereich eingeschlossen. Dies soll in erster Linie dazu dienen, praxisrelevante Ergebnisse präsentieren zu können. Neben ausgedehnten Tumoren oder besonders schwierigen Lokalisationen sind kleine noduläre BZK ein häufiger Grund für dermatochirurgische Eingriffe. Für diese Tumoren stehen verschiedene Therapiemöglichkeiten zur Verfügung. Von besonderer Bedeutung ist in der Therapieplanung die Frage, ob die aufwendigere Technik der mikrographisch gesteuerten Operation bei diesen Tumoren aufgrund der geringeren lokalen Rezidivraten gerechtfertigt ist. Alternative gering-invasive Operationstechniken sollte insbesondere bei Patienten in fortgeschrittenem Alter sowie bei schwerwiegenden Nebenerkrankungen diskutiert werden. In vielen Fällen ist diesen Patienten ein mehrtägiger stationärer Aufenthalt nicht zumutbar oder wird von den Patienten bzw. ihren Angehörigen nicht gewünscht. Therapieoptionen mit geringer Invasivität und der Vermeidung einer Hospitalisierung können für ausgewählte Patientengruppen wertvolle Alternativen darstellen. Lubeek et al. konnten beispielsweise bei jedoch kürzerer Nachbeobachtungszeit eine sehr geringe Rezidivrate von 6 % nach Kürettage und anschließender Elektrodesikkation von nodulären BZK zeigen [18]. Zu berücksichtigen ist jedoch das Risiko einer protrahierten Wundheilung und evtl. vermehrten Narbenbildung nach Elektrodesikkation.

Obwohl die Studiengruppe aus einer kleinen Anzahl von Patienten besteht, zeigen die vorliegenden Daten einen signifikanten Unterschied im Lokalrezidivverhalten der untersuchten Gruppen. Patienten, die mit 3‑D-Histologie-kontrollierten Exzisionen behandelt wurden, zeigten nach einem medianen Follow-up von 3,9 Jahren signifikant niedrigere Lokalrezidivraten im Vergleich zu Serienschnittexzisionen (*p* = 0,006). Die randomisierten prospektiven Studien von Smeets et al. und Mosterd et al. zeigten ebenfalls niedrigere Lokalrezidivraten für BZK nach mikroskopisch kontrollierter Operation [20, 28]. Allerdings wurde in beiden Studien keine Signifikanz erreicht, was möglicherweise auf die eingeschlossenen Tumoren zurückzuführen ist: Während unsere Arbeit ein Kollektiv von nodulären BZK mit einer Größenausdehnung bis 10 mm übersieht, wurden in die Arbeit von Smeets et al. und Mosterd et al. verschiedene Subtypen von BZK und auch Rezidivtumoren mit eingeschlossen [20, 28]. Bei Patienten, die in der vorliegenden Arbeit nur eine Kürettage des Tumors erhielten, zeigten 86,0 % kein Rezidiv. Dies ist vergleichbar mit den Ergebnissen von Kuijpers et al., die bei 17,6 % ein Rezidiv der BZK nach Kürettage und anschließender Kryotherapie fanden [13]. Es gab einen signifikanten Unterschied bezüglich der Lokalrezidivrate für die Kürettage- und die 3‑D-Gruppe, wobei die Lokalrezidive nach der Kürettage etwa 15-mal häufiger auftraten (14,0 % nach Kürettage gegenüber 0,9 % nach histologisch kontrollierten 3‑D-Exzisionen). Bei 24,4 % der Tumoren der 3‑D-Gruppe war eine Nachexzision erforderlich, bei weiteren 5,0 % der Patienten war eine zweite Nachexzision erforderlich. Bei einer relevanten Anzahl von Tumoren wurde nach der primären Exzision mit der 3‑D-Histologie diese nicht vollständig entfernt. Dies ist jedoch in erster Linie auf die typischerweise geringen Sicherheitsabstände der Erstexzision zurückzuführen. Die 3‑D-Histologie ermöglicht eine hautschonende Operation mit geringen initialen Sicherheitsabständen und der Möglichkeit, verbleibende Tumoranteile anatomisch orientiert darzustellen und selektiv nachzuresezieren.

Ein Vergleich der lokalen Rezidivraten nach Kürettage und Exzisionen mit anschließender serieller Schnitthistologie zeigt ebenfalls einen signifikanten Vorteil für die Patienten, die eine Exzision und Serienschnitthistologie erhielten (*p* = 0,005). Ein lokales Rezidiv wurde nach R1-Resektion in 25,0 % in der Serienschnittgruppe und 23,5 % in der Kürettagegruppe beobachtet und zeigte damit keinen relevanten Unterschied. In der 3‑D-Gruppe traten Lokalrezidive nur bei 0,9 % der Tumoren auf. Dies unterstreicht die Bedeutung einer vollständigen Entfernung des Tumors und ist mit den Ergebnissen von Mohs’ Chirurgie vergleichbar [16, 33]. Der evidente Vorteil niedriger Rezidivraten muss jedoch in Relation zum Aufwand bei weniger aggressiven BZK-Subtypen gesehen werden. Bei Patienten, die eine 3‑D-histologisch kontrollierte Exzision erhielten, gab es deutlich weniger Lokalrezidive, jedoch waren mehr Nachexzisionen erforderlich. Darüber hinaus erfordert mikrographisch kontrollierte Chirurgie speziell qualifiziertes Personal, ausreichende Infrastruktur hinsichtlich histologischer Aufarbeitung sowie Kommunikation zwischen Operateur und Histologen. Dies schlägt sich auch in höheren Kosten für mikrographisch kontrollierte Operationen nieder, wie bei der Operation von Mohs gezeigt werden konnte [20, 28]. Die Verwendung von Serienschnitten als histologische Aufbereitungsoption nach der Exzision ist also mit einem geringen Aufwand hinsichtlich histologischer und operativer Strukturen verbunden, zeigt aber höhere lokale Rezidivraten (*p* = 0,006). Die Lokalrezidivrate von 5,5 % in unserem Kollektiv ist vergleichbar mit der publizierten Literatur [3, 24, 27].

Im Gegensatz zu 3‑D-histologisch kontrollierten Exzisionen, die häufig aufwendigere Wundverschlusstechniken erfordern, stellt die Kürettage eine wenig komplexe chirurgische Behandlungsmöglichkeit dar. Diese Therapieoption hat gegenüber nichtchirurgischen Therapien auch den Vorteil, dass eine histologische Aufarbeitung möglich und einfach durchzuführen ist. Die Kürettage zeigt außerdem eine geringere Lokalrezidivrate als topische Therapien mit Imiquimod oder PDT [2, 4, 9, 12, 22, 23, 29–31, 35, 36]. Dies gilt auch, wenn exophytische Tumoranteile vor der PDT entfernt wurden [11, 29]. Bei der Anwendung von topischem Imiquimod (5 %) wurde eine vollständige histologische Entfernung von nodulären BZK <10 mm Durchmesser in 72,0 % berichtet, wobei 92,0 % der Patienten Nebenwirkungen berichteten [9]. In einer kleineren Kohorte konnten Wu et al. zeigen, dass topisches Imiquimod nach Kürettage in 94,0 % zu einer histologisch gesicherten Tumorfreiheit führte [36]. Dieses Vorgehen kann bei Patienten mit Komorbiditäten oder eingeschränkter Bereitschaft zur (stationär durchgeführten) Exzision und anschließendem Wundverschluss in Betracht gezogen werden. Eine prospektive Studie von Mosterd et al. verglich PDT mit 5‑Aminolävulinsäure mit der Exzision von BZK und beobachtete 30,3 % Lokalrezidive nach PDT [21]. Nach einem Follow-up von 3 Jahren berichteten Roozeboom et al. hochsignifikant bessere Ergebnisse für topisches Imiquimod im Vergleich zu MAL(Methylaminolevulinat)-PDT und topischem Fluoruracil zur Therapie superfizieller BZK [23]. Bemerkenswert ist auch, dass das ästhetische Ergebnis nach der Kürettage mit den nichtinvasiven Methoden vergleichbar ist [9, 29]. Ferner konnte gezeigt werden, dass sowohl Kürettage als auch Exzision mit anschließendem Verschluss zu guten ästhetischen Ergebnissen führen. Die Zufriedenheit mit dem ästhetischen Ergebnis war vergleichbar mit publizierten Daten zur Patientenzufriedenheit nach Defektverschlüssen im Gesichtsbereich [26]. Die Ergebnisse nach Kürettage wurden von den Patienten in 85,0 % als „exzellent“ oder „gut“ bewertetet.

Die Kürettage kleiner nodulärer BZK ist daher eine gute Alternative zur medikamentösen Therapie. Diese Methode ist leicht zu erlernen, zeitsparend und mit geringem Materialaufwand verbunden. Für frühe Formen von BZK oder Tumoren mit begrenzter Größe steht eine breite Palette chirurgischer Therapiemöglichkeiten zur Verfügung [8, 13, 14, 20, 28]. Je nach verfügbaren Ressourcen, Allgemeinzustand des Patienten bzw. Komorbiditäten und Lokalisation des Tumors sollte die Therapie nach einer strengen Risiko-Nutzen-Analyse ausgewählt werden. Für die Auswahl der geeigneten Therapieoption spielt neben der Verfügbarkeit von Ausstattung und geschultem Personal auch die Sinnhaftigkeit von wiederholten Reoperationen mit komplexem Wundverschluss bei einem immer älter werdenden Patientenkollektiv eine Rolle. In der vorliegenden Studie konnten wir zeigen, dass ein hoher Anteil der Kürettage kleinerer nodulärer BZK zu guten ästhetischen Ergebnissen mit vergleichsweise niedrigen Lokalrezidivraten führt. Die Kürettage erlaubt nicht nur eine histologische Aufarbeitung des Gewebes, sondern zeigt auch einen geringeren Anteil an Rezidiven als topische Therapien oder PDT. Je genauer histologische Kontrollen möglich sind, desto geringer ist die zu erwartende Lokalrezidivrate. Bei schwieriger Lokalisation oder einem Lokalrezidiv ist die 3‑D-histologisch kontrollierte Chirurgie auch bei BZK von begrenzter Größe eine etablierte Therapieoption, die mit einer sehr geringen Lokalrezidivrate einhergeht und in Betracht gezogen werden sollte.

## Fazit für die Praxis

- Basalzellkarzinome stellen als größte Gruppe der epithelialen Hauttumoren eine häufige Indikation für dermatochirurgische Eingriffe dar. Dabei ist die Operation weiterhin die Therapie der Wahl; hierfür stehen verschiedene Techniken zur Verfügung.
- Die 3‑D-Histologie ist mit geringen Lokalrezidivraten assoziiert, erfordert jedoch entsprechende Infrastruktur.
- Die Kürettage stellt eine einfach durchführbare Therapieoption mit der Möglichkeit einer histologischen Sicherung dar.
- Um eine Hospitalisierung bei ausgewählten Patientengruppen zu vermeiden, sollte diese Technik als Therapieoption berücksichtigt werden.
